# Clinical characteristics of Long COVID patients presenting to a dedicated academic post-COVID-19 clinic in Central Texas

**DOI:** 10.1038/s41598-023-48502-w

**Published:** 2023-12-11

**Authors:** Rija Aziz, Nadia Siles, Mary Kelley, Dennis Wylie, Esther Melamed, W. Michael Brode

**Affiliations:** 1 Department of Internal Medicine, Dell Medical School, University of Texas at Austin, Austin, USA; 2 Department of Neurology, Dell Medical School, University of Texas at Austin, Austin, USA

**Keywords:** Health care, Viral infection, Epidemiology

## Abstract

Post-acute sequelae SARS-CoV-2 (PASC), also known as Long COVID, is a complex and widely recognized illness with estimates ranging from 5 to 30% of all COVID-19 cases. We performed a retrospective chart review of patients who presented to a dedicated Post-COVID-19 clinic between June 2021 and May 2022. The median patient age was 44.5 years, 63.5% patients were female, and patients presented at a median of 10.4 months from acute COVD-19 infection. 78% self-identified their race as white, and 21% identified as Latino ethnicity. During the acute COVID-19 infection, 50% of patients experienced moderate disease severity and 10.5% were hospitalized. The top three co-morbid conditions prior to SARS-CoV-2 infection included mental health conditions, hypertension and asthma. Patients reported a median of 18 new symptoms following COVID-19 illness, the most common were fatigue (89%), forgetfulness or “brain fog” (89%), and difficulty concentrating (77%). MoCA (Montreal Cognitive Assessment) assessment demonstrated that 46% had mild cognitive dysfunction. PHQ-9 (Patient Health Questionnaire) testing revealed 42% had moderate to severe depression, and 38% had moderate to severe anxiety on the GAD-7 (Generalized Anxiety Disorder) assessment. Symptom burden was similar across gender, age, and initial disease severity. PASC patients presenting to an academic Post-COVID-19 clinic experienced numerous multisystem symptoms and functional impairment, independent of the initial COVID-19 disease severity.

## Introduction

Post-acute sequelae SARS-CoV-2 (PASC) is a complex and now widely recognized illness with estimates ranging from 5 to 30% of all COVID-19 cases^1–4^, and 37–76% of patients hospitalized with COVID-19^5,6^. The World Health Organization (WHO) defines PASC as post-COVID-19 conditions “usually 3 months from the onset of COVID-19 with symptoms that last for at least 2 months”^7^, although there is no consensus definition, diagnostic criteria, or reliable test for PASC. Over 203 symptoms in 10 organ systems have been identified in PASC^8^, with most studies identifying the most common symptoms as fatigue, post-exertional malaise, anosmia/ageusia and neurocognitive dysfunction^4,8,9^.

Recent data suggest that the risk of developing PASC may be decreasing with later SARS-CoV-2 variants, with one study demonstrating that 11% of Delta cases resulted in PASC compared to 4.5% of Omicron cases^10^, although the exact risk of PASC with ongoing variants is unknown. Vaccination has consistently been shown to reduce the risk of developing PASC^10–12^, although recently a large study found that vaccinations primarily reduce the risk by 15%^13^. It is estimated that 4 million people are out of the work force due to PASC and generate an addition $544 billion in healthcare costs each year^14^. Therefore, it is anticipated that PASC diagnoses and demand for specialized clinical services will continue to grow as variants continue to circulate.

Post-COVID-19 clinics have been created in most states across the United States to meet the demands of the growing PASC patient population, and Survivor Corps, a patient advocacy and support organization, has compiled a registry of more than 245 Post COVID Care Centers that have been opened in the United States^15^. These clinics vary widely in the provided services, ranging from individual physical therapy practices to academic medical centers with multispecialty interprofessional teams^16^.

Most studies have described PASC in large retrospective studies from electronic medical records (EMRs), databases, or cohort studies through surveys relying on patient responses. Few studies have been published investigating the characteristics of patients seeking medical care at dedicated outpatient PASC clinics^17,18^. In this study, we present a cohort of 252 patients who underwent evaluation at an academic tertiary center at Dell Medical School at the University of Texas at Austin, providing a comprehensive analysis of patient demographic factors, PASC symptomatology, co-morbid conditions, and clinical evaluations. Through this investigation, we aim to characterize the population of PASC patients seeking specialized medical care, which may inform the development of tailored PASC services and enhance the capacity of dedicated clinics to meet this escalating need.

## Results

In our multidisciplinary clinic, patients are initially evaluated by a general internist or advanced care provider to develop a comprehensive rehabilitation plan. Internal referrals are available for mental health counseling, neuroimmunology and rheumatology. For further subspecialty care that is not directly part of the multidisciplinary team, patients are referred to a network of providers with an interest and expertise in PASC, including physical and occupational therapy, pulmonology, cardiology, and autonomic neurology among other specialties.

The median age of our cohort was 44.5 years (IQR (interquartile range) 22.3), with a median BMI of 27.4 (IQR 9.85). 94% of patients had a COVID-19 diagnosis determined by lab criteria (confirmed by either SARS-COV-2 PCR or nucleocapsid antibody testing) or by the clinical evaluation of a healthcare provider, with the remaining 6% reporting that their initial COVID-19 infection was self-diagnosed. Patients presented at a median of 10.4 (IQR 10.25) months from acute SARS-CoV-2 infection, and 26/248 (10.5%) were hospitalized during their acute infection. Table 1 summarizes the patient demographic characteristics, employment data, acute infection severity and co-morbidities.

**Table 1:** Characteristics of Patients at the Post-COVID-19 Program.

Variable	Number (Percentage)	Variable	Number (Percentage)
**Gender/Ethnicity**	Total (n)* = 252	Other public insurance (Including MAP)	7 (2.8%)
Male	88 (35%)	Private insurance	183 (73%)
Female	160 (63.5%)	Other Insurance	24 (9.5%)
Latino	54 (21.4%)	**Employment**	Total (n) = 252
Not Latino	189 (75%)	Current employment	Full-time employed: 143 (56.7%)
			Part-time employed: 14 (5.6%)
			Unemployed: 38 (15%)
Preferred not to answer	9 (3.6%)		Retired: 13 (5.2%)
			Other: 44 (17.5%)
**Race**	Total (n) = 252	Employment prior 03/2020	Full-time employed: 195 (77.4%)
			Part-time employed: 11 (4.4%)
			Unemployed: 17 (6.7%)
			Retired: 8 (3.2%)
			Other: 21 (8.3%)
Black	10 (4%)	PASC effect on ability to work	Total (n) = 226
			Not impacted ability: 64 (28.3%)
			Hours reduced due to PASC: 110 (48.7%)
			Hours reduced but not due to PASC: 5 (2.2%)
			Temporarily laid off/lost job: 47 (20.8%)
White	197 (78%)	**COVID-19 vaccination**	Total (n) = 249
Asian	10 (4%)	Vaccinated ^a^	205 (82.3%)
American Indian	4 (1.6%)	Vaccinated before COVID-19 infection	54 (26%)
Middle Eastern	4 (1.6%)	Vaccinated after COVID-19 infection	149 (72.7%)
			PASC symptoms improved: 27 (18.1%)
			No change in PASC symptoms: 88 (59%)
			PASC symptoms got worse: 33 (22%)
Native Hawaiian	1 (0.4%)	**Acute infection severity**	Total (n) = 252
Other race	11 (4.4%)	No symptoms	7 (2.7%)
Preferred not to answer	15 (6%)		
**Lifestyle factors**	Total (n) = 252	Mild	89 (35.3%)
Current Smoker	7 (2.8%)	Moderate	126 (50%)
Former Smoker	77 (31%)	Severe	21 (8.3%)
1–3 drinks/week	66 (26%)	Critical	9 (3.6%)
3–6 drinks/week	27 (10.7%)	**Pre-existing medical condition**	Total (n) = 252
> 6 drinks/week	14 (5.6%)	No pre-existing medical condition	73 (29%)
No use of alcohol	143 (57%)	Mental health condition	59 (23.4%)
Recreational drug use	22 (9%)	Hypertension	53 (21%)
**Education**	Total (n) = 252	Asthma	52 (20.6%)
Less than high school degree	4 (1.6%)	Migraine	44 (17.5%)
High school diploma or GED	54 (21.4%)	High cholesterol	42(16.7%)
College or professional degree	194 (77%)	Autoimmune disorder	33 (13.1%)
**Insurance**	Total (n) = 252	Diabetes Mellitus	23 (9.1%)
None/uninsured	5 (2%)	Fibromyalgia	21 (8.3%)
Medicaid or CHIP	6 (2.4%)	Cancer	14 (5.6%)
Medicare	26 (10.3%)		

*Total number of patients who responded to the question.

^a^203 said yes to being vaccinated out of which 198 provided a date of vaccination.

Prior to their referral to the Post-COVID-19 Program, 91% of patients reported undergoing evaluation by a primary care physician, with cardiology, neurology, and pulmonology emerging as the top three specialties seen. Table 2 provides further details on the healthcare providers from whom patients received care before their presentation to the clinic.

**Table 2:** Patients who sought care from healthcare professionals for PASC symptoms, Total Number (n) = 252.

Healthcare professional	Number (percentage)
Primary care physician	229 (91%)
Cardiologist	111 (44%)
Neurologist	74 (29.4%)
Pulmonologist	67 (26.6%)
Physical therapist	54 (21.4%)
Psychologist or social worker	51 (20.2%)
Rheumatologist	39 (15.5%)
Gastroenterologist	22 (8.7%)
Physical medicine	17 (6.7%)
Nephrologist	4 (1.6%)

Evaluation of health-related quality of life using the Patient-Reported Outcomes Measurement Information System (PROMIS) Global 10 v1.2 assessment demonstrated a mean T score of 37.9 and 37.5 for mental and physical health, respectively. The proportion of patients with “fair” or “poor” mental scores was 65%, and for physical scores it was 73% (Table 3). Assessment of cognitive dysfunction using the Montreal Cognitive Assessment (MoCA) demonstrated that 46% experienced mild cognitive dysfunction. Patient Health Questionnaire 9 (PHQ-9) screening for depression revealed 42% of patients had moderate, moderately severe, or severe depression. Furthermore, using the Generalized Anxiety Disorder 7 (GAD-7) assessment, we identified that 38% had moderate to severe anxiety, and on the Primary Care PTSD Screen for DSM-5 (PC-PTSD-5), 63% had a score of 1 or greater (Table 4). MoCA scores were lower in patients who were hospitalized (mean score 22.5) during the acute COVID-19 illness compared to patients who were not hospitalized (mean score 25.5. Wilcox test, *p*-value < 0.001). The spearman correlation coefficient between PHQ-9 and MoCA scores was -0.2 without controlling for age (p-value < 0.01). For social determinants of health, 19% of patients had an unmet health related social need, most frequently being unable to get “medicine or health care” when needed. In addition, 12% of patients endorsed housing insecurity.

**Table 3:** Categorization of patient population’s PROMIS scores:

	***Mean***	***Standard Deviation***	***Number (%) with scores “excellent”***	***Number (%) with scores “very good”***	***Number (%) with scores “good”***	***Number (%) with scores “fair”***	***Number (%) with scores “poor”***
PROMIS Mental T Score (total reported = 248)	37.9	9.05	10 (4%)	24 (10%)	53 (21%)	126 (51%)	35 (14%)
PROMIS Physical T Score (total reported = 248)	37.5	8.3	4 (2%)	11 (4%)	53 (21%)	79 (32%)	101 (41%)

**Table 4:** Breakdown of score assessments.

**MoCA scores**	*Normal ( > 25)*	*Mild (18–25)*	*Moderate (10–17)*	*Severe ( < 10)*	
Number (percentage): total 211	108 (51%)	97 (46%)	6 (3%)	0 (0%)	

**PHQ-9**	*Minimal (1–4)*	*Mild (5–9)*	*Moderate (10–14)*	*Moderately severe (15–19)*	*Severe (20–27)*
Number (percentage): total 221	71 (32%)	7 (3%)	33 (14.8%)	30 (13.5%)	30 (13.5%)

**GAD-7**	*Minimal (0–4)*	*Mild (5–9)*	*Moderate (10–14)*	*Severe (> = 15)*	
Number (percentage): total 221	107 (48.4%)	30 (13.6%)	43 (19.5%)	41 (18.6%)	

**PC-PTSD**	*Score of 0*	*Score of 1–3*	*Score of 4–5*		
Number (percentage): total 192	71 (37%)	67 (35%)	54 (28.1%)		

### Evaluation of review of systems in PASC patients

**Figure 1:**
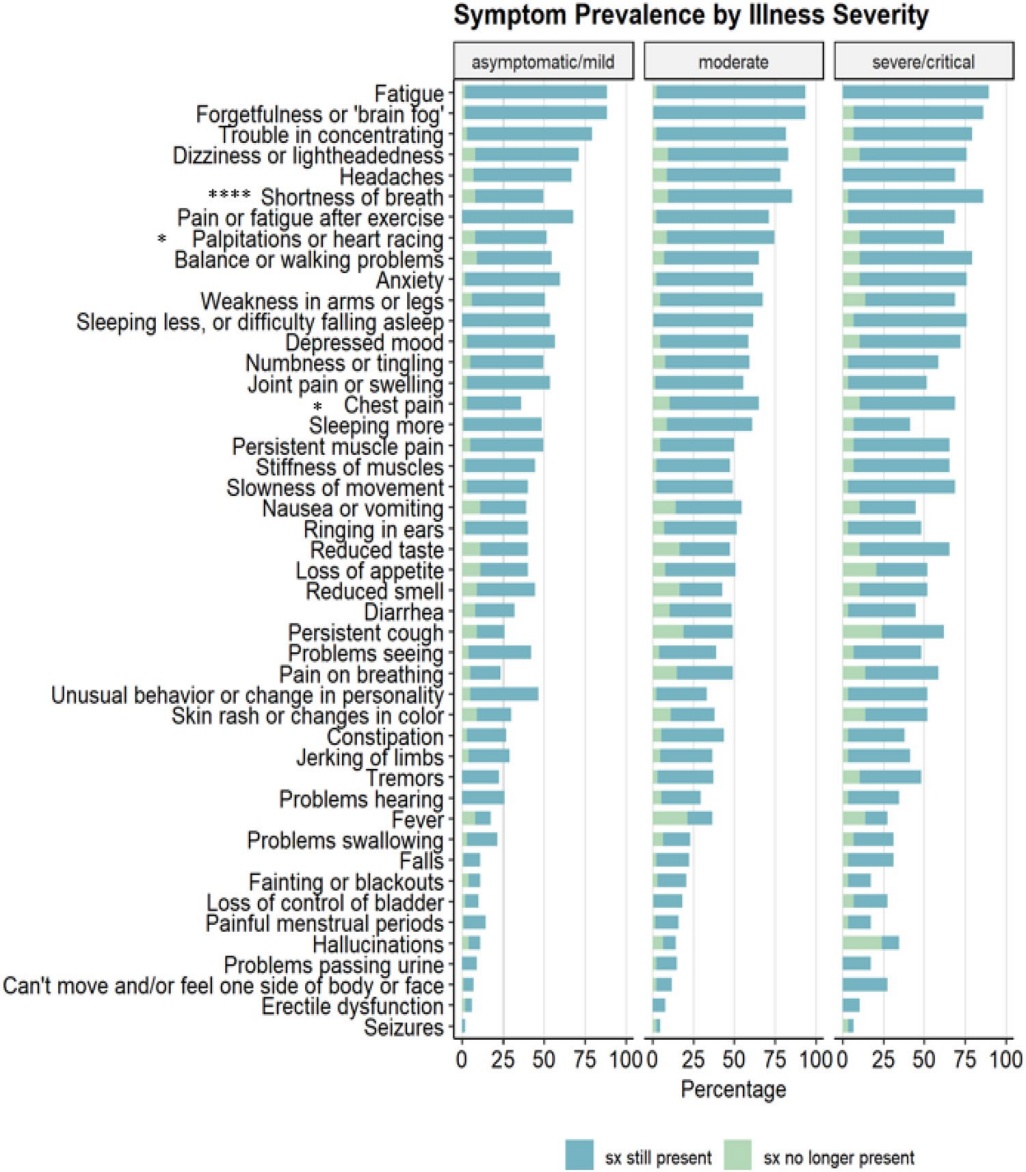
Symptom prevalence by illness severity. Bars represent the percentage of participants in their respective category who had experienced and/or continued to experience each symptom when they presented at the PASC clinic. The time from acute infection to PASC clinic presentation varies from patient to patient. Symptoms labeled with asterixes were significantly associated with illness severity. (Fisher exact test, * *p* ≤ 0.05, ** *p* ≤ 0.01, *** *p* ≤ 0.001, **** *p* ≤ 0.0001).

Symptom prevalence in 10 body systems (constitutional, cardiovascular, respiratory, rheumatologic, gastrointestinal, genitourinary, musculoskeletal, integumentary, neurological, and psychiatric) was estimated through a total of 46 surveyed symptoms. More than three fourths of all participants experienced constitutional and neurological symptoms while respiratory, psychiatric, cardiovascular, and rheumatologic symptoms were prevalent in greater than 50% of our cohort. The top 5 symptoms were fatigue [n = 225, (89%)], forgetfulness or ‘brain fog’ [n = 224, (89%)], trouble concentrating [n = 195, (77%)], dizziness or lightheadedness [n = 173, (69%)] and pain or fatigue after exercising [n = 172, (68%)]. Due to COVID-19 infection, participants experienced a median of 18 new symptoms (IQR 14) and a median of 1 resolved symptom (IQR 4) at the time of the initial clinic visit. 43% of patients reported less than 16 symptoms, 45% reported 16–30 symptoms, and 12% reported greater than 30 symptoms. Symptom prevalence was similar independent of the initial disease severity (Fig. 1). Additional symptom prevalence estimates are shown in Supplemental Figs. 1–5, categorized by age quantiles (1), sex (2), alcohol consumption (3), smoking status (4), and recreational drug use (5). The type and number of symptoms reported were similar among patients with different pre-existing conditions; however, using a logistic regression model, patients with pre-existing vascular disease were more likely to report more than 30 symptoms compared to fewer than 16 (OR 3.6, CI [1.49–9.03], *p*.adj = 0.0168).

## Discussion

Our results support the concept that PASC is a multifaceted chronic disease that can occur in individuals who were either hospitalized or non-hospitalized during the acute COVID-19 illness. Notably, nearly all patients in our cohort experienced some level of neurological or constitutional symptoms. Furthermore, most patients reported their health as fair to poor on the PROMIS Global 10 measure, indicating substantial and concerning levels of functional impairment and diminished health-related quality of life. Interestingly, we observed a high degree of similarity in PASC symptoms and clinical history between patients, suggesting that the underlying PASC pathophysiology may be shared between individuals rather than representing multiple discrete illnesses.

As seen in previous PASC studies, our patient population had a higher BMI, was middle aged, non-Hispanic white and consisted of a predominantly female cohort^4,19^. Our patients were also relatively younger than patients at risk of severe acute COVID-19 infection^20^. We found that the development of PASC symptoms was independent of the severity of the acute illness, as the majority (89.5%) of our patients were never hospitalized, which was similar to a study (Shanley et al.2022)^21^ where 82% of the cohort had mild or moderate initial illness. In part, this finding is reflective of the epidemiology of COVID-19, as the vast majority of patients do not require hospitalization. Given the young age of our cohort and that 29% of patients had no pre-existing medical conditions, research into identifying individual risk factors for the development of PASC is crucial. Based on these findings, an important conclusion from our study is that structuring PASC clinical services around a post-ICU outpatient model may only be appropriate for a minority of patients seeking PASC care.

Not surprisingly, we observed that patients’ ability to work was significantly impacted by PASC with an overall reduction in full time employment, an increase in part time employment, and a doubling of the unemployment rate. This finding has been supported by several other studies outlined in a recent review^22^, indicating a clear impact of PASC on the financial well-being of patients. Limited information is currently available regarding which specific symptoms of PASC may be contributing to the observed effect in employment. Possible explanations may include cognitive dysfunction, fatigue and pain experienced as evident in poor performances on MoCA and PROMIS assessments of our patient population, which may impede participation in the workforce.

In our study, we did not observe reliable improvement in PASC symptoms following vaccination. In a recent review evaluating 16 PASC studies, most patients with PASC reported symptomatic improvement following SARS-CoV-2 vaccination without a significant difference between the various types of SARS-CoV-2 vaccines, though the strength of these findings is limited by a small proportion of participants reporting either worsening of symptoms or no change in the symptoms^23^. More research is needed to further elucidate SARS-CoV-2 vaccine effects in PASC patients and identify biomarkers to determine which PASC patients may or may not have a therapeutic benefit from vaccination.

The symptoms of fatigue, “brain fog” and post-exertional malaise were reported by our patients at a very high frequency, aligning with the observed similarities between Myalgic Encephalomyelitis/Chronic Fatigue Syndrome (ME/CFS) and PASC^24^. The diagnostic criteria for ME/CFS include severe fatigue, post-exertional malaise, and unrefreshing sleep, in addition to either cognitive impairment or orthostatic intolerance^25^. Although we did not directly screen for ME/CFS, based on survey responses, we estimate that at least 60% of our cohort would meet the diagnostic criteria. This estimate is derived from patients endorsing concurrent ROS symptoms of fatigue, pain or fatigue after exercising, sleep disturbance, along with one additional symptom such as brain fog, trouble concentrating, dizziness/lightheadedness, or palpitations. This finding reinforces the need for dedicated screening and additional research on the intersection between PASC and ME/CFS.

Despite the almost universal reporting of subjective neurocognitive complaints, we found that about half of our patient cohort had a score within the normal range on the MoCA, and the other half was noted to have mild or worse cognitive dysfunction. In other studies, the odds of having an abnormal MoCA score compared to normal was elevated (5.84) with high premorbid vulnerability NHS score and more severe pulmonary disease^26^. We observed a similar finding in our study, that MoCA scores were lower in patients who had severe or critical illness requiring hospitalization, compared to the group that was not hospitalized. In a systematic review and meta- analysis of the cognitive effects of COVID-19, impairment was found in executive function, attention and memory collectively referred to as “brain fog”^27,28^. Similarly, in our study, most patients endorsed forgetfulness or ‘brain fog’ (89%), and deficits in concentration and attention (77%).

In a recent study using MoCA screening in patients with PASC, depression was shown to be the strongest predictor of persistent cognitive complaints^29^. Interestingly, we also observed that the spearman correlation coefficient between PHQ-9 and MoCA scores was -0.2 without controlling for age. It is important to note that the PHQ-9 screens for both psychological and physiologic symptoms of depression which includes deficits in concentration, changes in sleep, and poor energy. Therefore, differentiating depression symptoms from the neurocognitive effects of PASC is difficult, and the overlap may account for the inverse relationship between MoCA and PHQ-9 scores. A prior study has found that the course of depression and anxiety was more severe in COVID-19 survivors in those with prior psychiatric history than those without^30^. Further research is necessary to determine if interventions targeted to the affective symptoms of PASC could alleviate the cognitive deficits, given the high co-occurrence of depression and anxiety in our cohort.

The pathophysiology of PASC remains poorly understood, though a key hypothesis posits that it is caused by immune dysregulation, and PASC appears to share similarities with autoimmune conditions^31^. Studies have suggested autoimmunity as a key factor in PASC, with reports of persistently positive anti-nuclear antibodies (ANAs) associated with ongoing symptoms and inflammation in COVID-19 survivors^32^. Additionally, antibodies targeting angiotensin-converting enzyme 2 (ACE2) have been linked to a pro-inflammatory state in PASC^33^. In our study, we observed a multitude of constitutional symptoms, and the proposed immune dysregulation might explain the basis of these diverse manifestations in PASC patients. Other hypotheses propose that neurological symptoms might be due to direct virus-related CNS damage and/or indirect damage due to ongoing autoimmune inflammation, or sequelae of hypoxia^34^. Further research is warranted to identify precise biomarkers for PASC toward developing tailored therapeutic approaches.

Lifestyle factors, such as smoking and alcohol abuse have been previously implicated in slower recovery of PASC symptoms in other studies^19^. In our study, 66% of patients reported that they were neither current nor former smokers and 57% reported that they had 0 alcohol drinks per week. The disparity in our findings compared to prior studies could be attributed to differences in our sample population, potentially comprising more health-conscious individuals. Moreover, our reliance on self-reporting of substance use might lead to underestimation in self-reports, which could also contribute to the variations observed.

Regarding healthcare utilization, most patients initially sought care from their primary care providers, and many were referred to subspecialists before visiting our clinic. The high rate of subspecialty referrals likely stems from the unclear pathophysiology of PASC, the complex nature of patients' clinical presentation, and the challenges associated with diagnosing and treating PASC. Consequently, there is a clear need for consolidated care within multidisciplinary Post-COVID-19 clinics, capable of addressing the intricate diagnostics and treatment of patients while facilitating research evaluations to gain a more comprehensive understanding of PASC pathophysiology.

The strengths of our study included a large cohort with a detailed description of clinical PASC symptoms through standardized clinical assessments and evaluation of patient demographic factors. Notably, our response rate was 100%, which is greater than other PASC clinic studies^35^, and demonstrates the feasibility of integrating comprehensive research evaluations in PASC clinical care. Moreover, our assessments were conducted at a median of 10.5 months from acute infection, providing valuable insights into the persistence of PASC compared to studies with assessments at shorter durations^36,37^. We also specifically focus on patients who sought care for PASC regardless of their hospitalization history.

There were also several limitations in our study. First, this study was conducted at a single clinic at a tertiary academic center that drew from a single geographic region of Central Texas and consisted of patients who actively sought specialized PASC care without a control group, which limits the generalizability of the study findings. Survey responses relied on patient recall and answers may be less reliable particularly in those with cognitive complaints, introducing recall bias. In addition, none of the questions in the physician-designed standard of care questionnaire were mandatory, leading to missing data. We primarily used PHQ-9, GAD-7, and PC-PTSD-5 to compare our results with those of the general population, as these tools were already a standard part of care at our institution. While these general screening tools are utilized widely^38,39^, other instruments such as the Hospital Anxiety and Depression Scale (HADS) and Impact of Events Scale 6 (IES-6) may be potentially more effective at isolating affective symptoms directly related to COVID-19 illness. Additionally, our patient population consisted primarily of White female, non-Latino Americans, with Latinos representing the second-largest group. The overrepresentation of patients with private insurance and college education likely reflects the healthcare-seeking behavior and higher socioeconomic capacity of this group to obtain care, rather than the prevalence of PASC symptoms in the general population. Our findings underscore the importance that dedicated PASC clinics take proactive measures to enroll underserved patients and establish the capacity to provide inclusive services for those currently lacking access to specialized care.

## Methods

### Study design and setting

The University of Texas at Austin Institutional Review Board (IRB) reviewed the study protocol for this retrospective chart review and determined that this protocol met the criteria for exemption from IRB review under 45 CFR 46.104 (4) secondary research on data or specimens. The IRB approved the request to collect and use protected health information (PHI) for research purposes. In addition, the IRB waived the requirement to obtain subject authorization for use and disclosure of PHI and waived the requirement of informed consent to obtain prior authorization of the individuals affected under 45 CFR 164.512. All methods were carried out in accordance with relevant guidelines and regulations of the University of Texas at Austin IRB.

The study population comprised of patients who presented to the Post-COVID-19 Program between June 2021 and May 2022, with an initial COVID-19 illness onset between November 2019 and January 2022. Patients were required to be 18 years or older to be included in the study. To become a patient at the clinic, individuals were required to have reached a minimum of 12 weeks from the onset of their illness. Documentation of a positive COVID-19 test was not required to receive services, although all patients were screened by a dedicated nurse to validate that their clinical history was consistent with a prior COVID-19 infection, and the symptoms are likely associated with PASC and not an obvious alternative etiology.

### Measurements

Prior to their initial appointment at the Post-COVID-19 Program, patients completed a physician-designed standard of care questionnaire via Research Electronic Data Capture (REDCap). Categorization of the severity of the acute SARS-CoV-2 was determined using the National Institute of Health (NIH) definitions^40^. The review-of-symptoms questionnaire list was adapted from the World Health Organizations Global COVID-19 Clinical Platform Case Report Form for Post COVID Condition^41^. For symptom response choices, we interpreted “Yes, the symptom is still present” and “Yes, the symptom comes and goes” as a positive for having the symptom, a negative answer for "Yes, but not present anymore," "Unknown," and un-answered responses, as indicated in the ROS instructions.

The questionnaire included the PROMIS Global 10 v 1.2 score, which was used to collect patients' perceptions of their health. This item is useful in determining overall well-being and has been shown to reliably predict healthcare utilization and subsequent mortality^42^. The PROMIS measures were interpreted using T-score cutoffs recommended for the general adult reference population^43^.

We also administered MoCA scores to assess cognitive dysfunction^27^ during patients’ initial visits. Additionally, the PHQ-9, a self-administered diagnostic instrument, was used to screen for depression^44^. Anxiety symptoms were assessed using the GAD-7, a self-reported questionnaire for screening and severity of anxiety^45^. We screened for PTSD using the PC-PTSD-5 screening tool; a 5-item screening questionnaire, designed for use in primary care settings, with a cut-off score of 3 being optimally sensitive to suggest probable PTSD^46^. All these assessments were completed on paper or tablets and subsequently uploaded into REDCap. These assessments were encouraged but optional to patients, some declined or were not able to complete these assessments which resulted in missing data.

### Data analysis

Participant demographics were reported as mean, median, or frequency (percentage). Those with missing data were excluded from the analysis of the variable of interest. Comparisons between groups were done using Wilcoxon signed-rank test or chi-square tests when appropriate. The Fisher’s Exact Test was used to assess the association between symptom burden and patient characteristics. The calculated *p* values were corrected for multiple hypothesis testing using the Benjamini and Hochberg method and the double FDR adjustment when appropriate. All graphing and analyses were conducted using R 4.1.0. and Microsoft Excel Version 2019. Symptom prevalence was investigated by identifying the presence or absence of 46 PASC symptoms as listed in the supplementary tables.

## Conclusion

The majority of PASC patients in our cohort had mild to moderate acute COVID-19 disease and did not require hospitalization. Most patients experienced ongoing symptoms for the past 10 months, with significant functional impairment, indicating that PASC is a long-term debilitating illness for affected patients. Notably, there was significant symptom overlap between patients, suggesting likely overlapping pathophysiology across PASC. Given the complexity of diagnosis and treatment of PASC, multispecialty clinics are required to meet the needs of the PASC patient population with integration of research studies toward better understanding long-term health consequences of PASC and developing evidence based PASC treatments.

### Supplementary Information

Supplementary Information.

## Data Availability

The datasets generated and/or analyzed during the current study are not publicly available because although the data is de-identified, it contains comprehensive assessments of physical and mental health of individual participants. The de-identified datasets are available from the corresponding author on reasonable request.
