# Supplementary material for: Clinical characteristics of Long COVID patients presenting to a dedicated academic post-COVID-19 clinic in Central Texas
Source: Sci Rep. 2023 Dec 11;13:21971. doi: 10.1038/s41598-023-48502-w (PMC10713530; doi:10.1038/s41598-023-48502-w)
Supplement: Supplementary file 1 — Supplementary Information. [file 41598_2023_48502_MOESM1_ESM.docx]

| 1. Anxiety | 9. Painful menstrual periods | 17. Hallucinations | 25. Pain on breathing |
| --- | --- | --- | --- |
| 1. Unusual behavior or change in personality | 10. Dizziness or lightheadedness | 18. Headaches | 26.Palpitations or heart racing |
| 1. Balance or walking problems | 11. Erectile dysfunction | 19. Jerking of limbs | 27. Persistent cough |
| 1. Can’t move and/or feel one side of body or face | 12. Fainting or blackouts | 20. Joint pain or swelling | 28. Problems hearing |
| 1. Chest pain | 13. Falls | 21. Loss of appetite | 29. Persistent muscle pain |
| 1. Constipation | 14. Fatigue | 22. Loss of control of bladder | 30. Pain or fatigue after exercise |
| 1. Depressed mood | 15. Fever | 23. Nausea or vomiting | 31. Problems passing urine |
| 1. Diarrhea | 16. Forgetfulness or “brain fog” | 24. Numbness or tingling | 32. Problems seeing |
| 33. Problems swallowing | 34. Reduced smell | 35. Reduced taste | 36. Ringing in ears |
| 37.Seizures | 38. Shortness of breath | 39. Skin rash or changes in color | 40. Slowness of movement |
| 41. Sleeping more | 42. Sleeping less, or difficulty falling asleep | 43. Stiffness of muscles | 44. Tremors |
| 45. Trouble in concentrating | 46. Weakness in arms or legs |  |  |

**Supplementary table 1: Review of symptoms due to PASC**


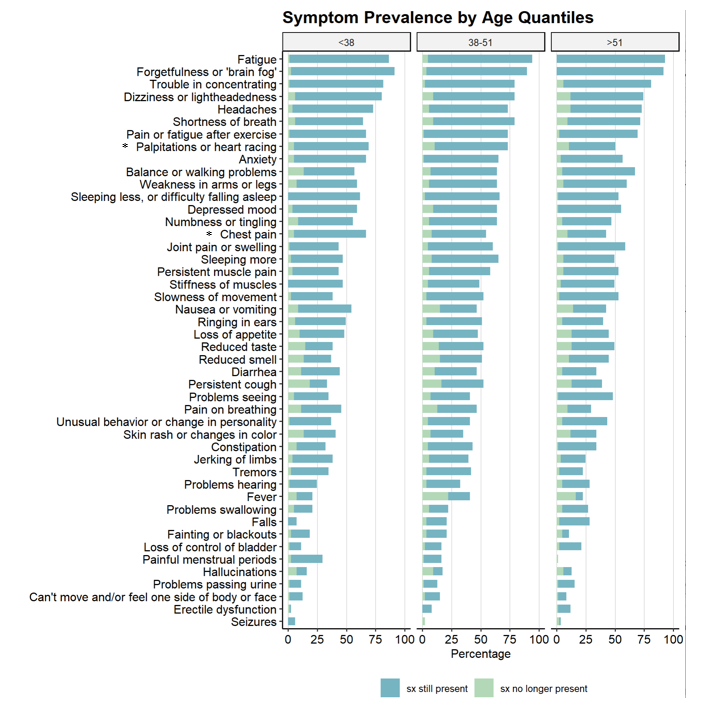


**Supplementary Figure 1.**

**Supplementary Figure 1: Symptom prevalence by age.**  Bars represent the percentage of participants in their respective category who had experienced and/or continued to experience each symptom when they presented at the PASC clinic. The time from acute infection to PASC clinic presentation varies from patient to patient. Symptoms labelled with asterixis were significantly associated with illness severity. (Fisher exact test, * p ≤ 0.05, ** p ≤ 0.01, *** p ≤ 0.001, **** p ≤ 0.0001).


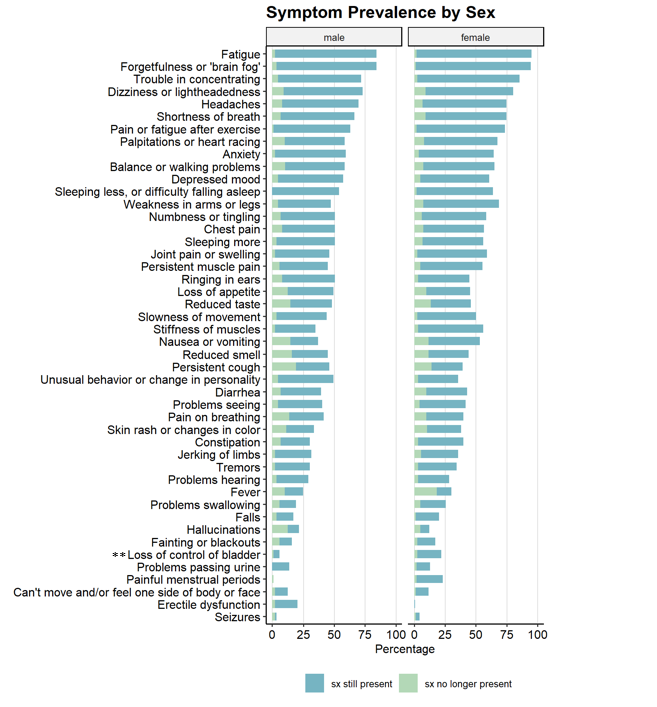


**Supplementary Figure 2**

**Supplementary Figure 2: Symptom prevalence by sex.**  Bars represent the percentage of participants in their respective category who had experienced and/or continued to experience each symptom when they presented at the PASC clinic. The time from acute infection to PASC clinic presentation varies from patient to patient. Symptoms labelled with asterixis were significantly associated with illness severity. (Fisher exact test, * p ≤ 0.05, ** p ≤ 0.01, *** p ≤ 0.001, **** p ≤ 0.0001).


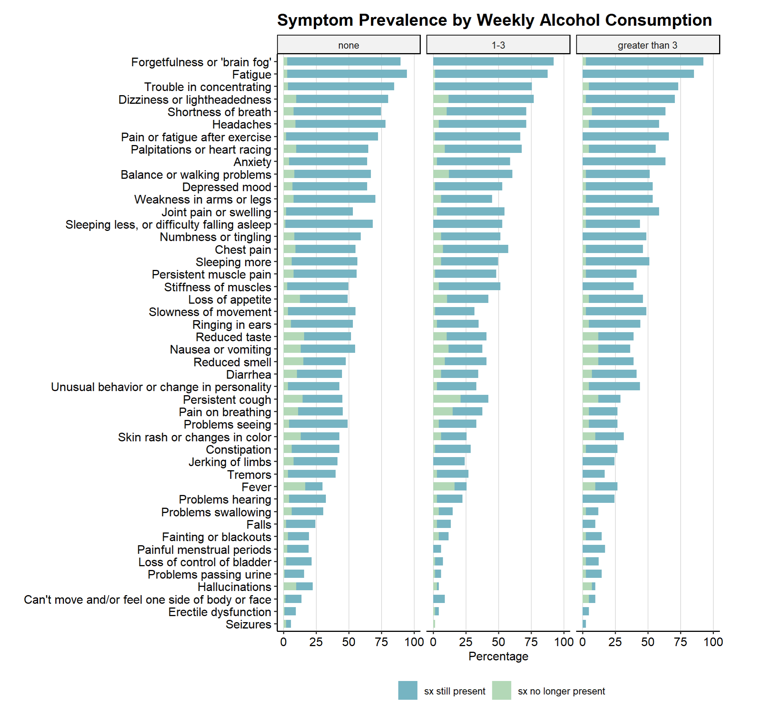


**Supplementary Figure 3**

**Supplementary Figure 3: Symptom prevalence by alcohol consumption.**  Bars represent the percentage of participants in their respective category who had experienced and/or continued to experience each symptom when they presented at the PASC clinic. The time from acute infection to PASC clinic presentation varies from patient to patient. Symptoms labelled with asterixis were significantly associated with illness severity. (Fisher exact test, * p ≤ 0.05, ** p ≤ 0.01, *** p ≤ 0.001, **** p ≤ 0.0001).


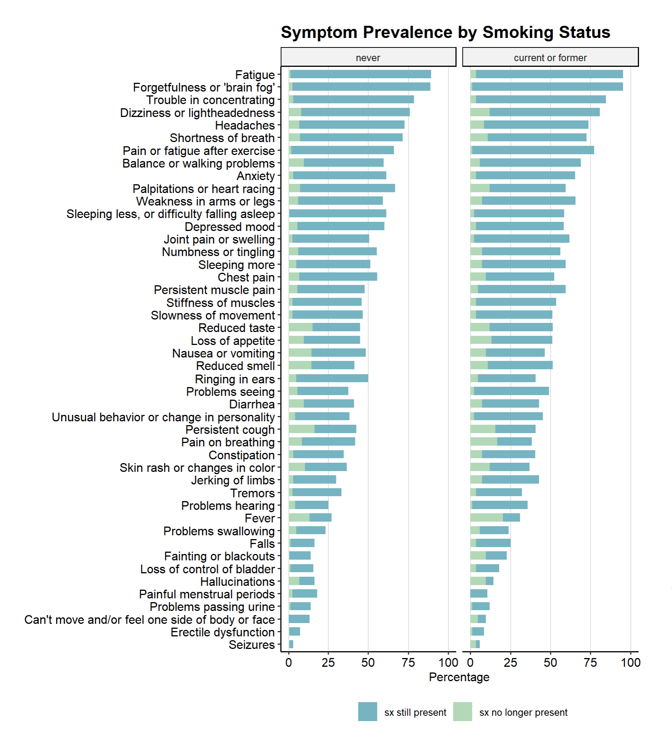


**Supplementary Figure 4**

**Supplementary Figure 4: Symptom prevalence by smoking status.**  Bars represent the percentage of participants in their respective category who had experienced and/or continued to experience each symptom when they presented at the PASC clinic. The time from acute infection to PASC clinic presentation varies from patient to patient. Symptoms labelled with asterixis were significantly associated with illness severity. (Fisher exact test, * p ≤ 0.05, ** p ≤ 0.01, *** p ≤ 0.001, **** p ≤ 0.0001).


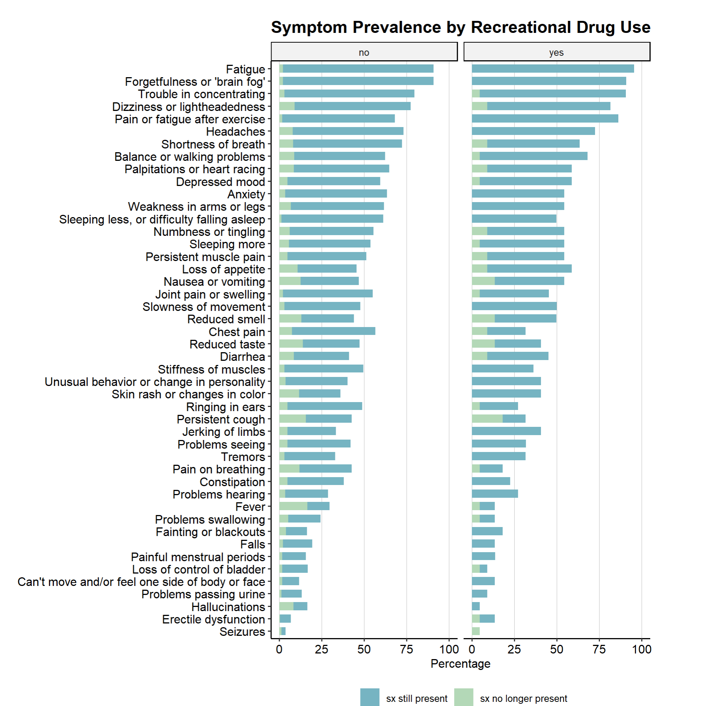


**Supplementary Figure 5**

**Supplementary Figures 5: Symptom prevalence by recreational drug use.**  Bars represent the percentage of participants in their respective category who had experienced and/or continued to experience each symptom when they presented at the PASC clinic. The time from acute infection to PASC clinic presentation varies from patient to patient. Symptoms labelled with asterixis were significantly associated with illness severity. (Fisher exact test, * p ≤ 0.05, ** p ≤ 0.01, *** p ≤ 0.001, **** p ≤ 0.0001).
